# Authors’ reply

**Published:** 2010

**Authors:** Vikash Kumar Singh, Jayesh V. Dhabalia, Girish G. Nelivigi, Mahendra S. Punia, Manav Suryavanshi

**Affiliations:** Department of Urology, LTMGH and LTMMC, Sion, Mumbai, India; 1 Department of Urology, KEM Hospital, Parel, Mumbai - 400 012, India

Dear Editor,

I appreciate Dr. Arvind P. Ganpule for his thoughtful comments[1] on our paper,[2] but we will like to clarify the queries raised. Firstly, this study has been conducted in Mumbai, a cosmopolitan city, catering population from all over India. So we have got an adequate number of healthy population from different parts of India (North, South, West and East). We agree that a multicenter study from different parts of India using subset of population will be more accurate. We have used corrected Qmax to prepare the nomogram and avoid fallacious peak in Qmax due to instrumental error.

We do not agree, as suggested, that baseline urine routine examination and USG is mandatory to identify healthy population. Healthy population is not required to be screened for asymptomatic infection and clinical implication of asymptomatic infection on uroflowrate is unknown. USG KUB was done in initial 100 patients; it was normal in the first 100 patients and it was discontinued in subsequent population as it was adding cost without much help in selection or exclusion of healthy population. There are many studies on nomogram preparation not using urine routine microscopy and USG KUB as screening for healthy population.[3–5]

This original study was presented in USICON 2009 and was awarded the Prof C K P Menon prize after being reviewed by USI chairpersons and judges. The error in total number of patients (mentioned 1011 in place of 1017) occurred because of typing mistake and it does not affect our nomogram preparation and statistical analysis. This minor mistake had happened after editing and typing in manuscript format that was not there in my presentation and sent to the USI before its acceptance as consideration for the Prof C K P Menon prize. So we still think this original study was worthy enough to be accepted for the prize and its acceptance for publication in an indexed journal (IJU).

As for the last query, the article published by Ganpule *et al*.[6] is not the study for drawing Uroflowmetry nomogram. We were unable to find this article after putting “Uroflowmetry nomogram” as search option in Pubmed, hence we missed this article. Secondly, this study is conducted in patients with LUTS and community patients of a specific age group (mean age 62.1 years, SD 9.5, range 40-82, male patients). The aim was to know the prevalence of LUTS in community and correlation between LUTS, age, prostate volume and quality of life, but in our study we have taken healthy population including all age groups and different gender and prepared the Uroflowmetry nomogram. Therefore, we totally disagree with the opinion drawn that earlier study concluded the same as we have come out with.
